# Implementing an intensive care registry in India: preliminary results of the case-mix program and an opportunity for quality improvement and research

**DOI:** 10.12688/wellcomeopenres.16152.2

**Published:** 2020-10-21

**Authors:** Neill K. J. Adhikari, Rajeshwari Arali, Udara Attanayake, Sampath Balasundaram, Abi Beane, Vijay Chakravarthy, Niyaz Channanath Ashraf, Sri Darshana, Dedeepiya Devaprasad, Arjen M. Dondorp, Robert Fowler, Rashan Haniffa, Pramodya Ishani, Augustian James, Issrah Jawad, Devachandran Jayakumar, Chamira Kodipilly, Rakesh Laxmappa, Kishore Mangal, Ashwin Mani, Meghena Mathew, Sristi Patodia, Rajyabardhan Pattnaik, Dilanthi Priyadarshini, Mathew Pulicken, Ebenezer Rabindrarajan, Pratheema Ramachandran, Kavita Ramesh, Usha Rani, Suchitra Ranjit, Ananth Ramaiyan, Nagarajan Ramakrishnan, Lakshmi Ranganathan, Thalha Rashan, Raymond Dominic Savio, Jaganathan Selva, Bharath Kumar Tirupakuzhi Vijayaraghavan, Swagata Tripathy, Timo Tolppa, Ishara Udayanga, Ramesh Venkataraman, Deepak Vijayan

**Affiliations:** 1 Indian Registry of IntenSive care, IRIS, Chennai, India; 2 Critical Care Medicine, Sunnybrook Health Sciences Centre, Toronto, Canada; 3 Critical Care Medicine, Apollo Children's Hospital, Chennai, India; 4 Network for Improving Critical care Systems and Training, NICST, Colombo, Sri Lanka; 5 Chennai Critical Care Consultants, Chennai, India; 6 Critical Care, Mahidol Oxford Tropical Medicine Research Unit, Bangkok, Thailand; 7 Critical Care Medicine, Apollo Hospitals, Chennai, India; 8 Critical Care Medicine, Nanjappa Hospital, Shimoga, India; 9 Critical Care Medicine, Eternal Hospitals, Jaipur, India; 10 Critical Care Medicine, Ispat General Hospital, Rourkela, India; 11 Critical Care Medicine, Pushpagiri Hospital, Tiruvalla, India; 12 Critical Care Medicine, ABC Hospitals, Vishakapatnam, India; 13 Critical Care Medicine, Mehta Hospitals, Chennai, India; 14 Anaesthesia and Intensive Care Medicine, All India Institute of Medical Sciences, Bhubaneswar, India; 15 Critical Care Medicine, Kerala Institute of Medical Sciences, Thiruvananthapuram, India

**Keywords:** critical care, registries, India

## Abstract

**Background: **The epidemiology of critical illness in India is distinct from high-income countries. However, limited data exist on resource availability, staffing patterns, case-mix and outcomes from critical illness. Critical care registries, by enabling a continual evaluation of service provision, epidemiology, resource availability and quality, can bridge these gaps in information. In January 2019, we established the Indian Registry of IntenSive care to map capacity and describe case-mix and outcomes. In this report, we describe the implementation process, preliminary results, opportunities for improvement, challenges and future directions.

**Methods: **All adult and paediatric ICUs in India were eligible to join if they committed to entering data for ICU admissions. Data are collected by a designated representative through the electronic data collection platform of the registry. IRIS hosts data on a secure cloud-based server and access to the data is restricted to designated personnel and is protected with standard firewall and a valid secure socket layer (SSL) certificate. Each participating ICU owns and has access to its own data. All participating units have access to de-identified network-wide aggregate data which enables benchmarking and comparison.

**Results: **The registry currently includes 14 adult and 1 paediatric ICU in the network (232 adult ICU beds and 9 paediatric ICU beds). There have been 8721 patient encounters with a mean age of 56.9 (SD 18.9); 61.4% of patients were male and admissions to participating ICUs were predominantly unplanned (87.5%). At admission, most patients (61.5%) received antibiotics, 17.3% needed vasopressors, and 23.7% were mechanically ventilated. Mortality for the entire cohort was 9%.  Data availability for demographics, clinical parameters, and indicators of admission severity was greater than 95%.

**Conclusions: **IRIS represents a successful model for the continual evaluation of critical illness epidemiology in India and provides a framework for the deployment of multi-centre quality improvement and context-relevant clinical research.

## What is already known?

- The epidemiology of critical illness in low- and lower-middle-income countries (LMICs) is distinct from high-income countries in the types of illnesses that brings patients into ICUs, in resource availability and access to care, in funding models for healthcare and in the burden of antimicrobial resistance.
- There is limited data from India and other LMICs on case-mix and outcomes for critical illness, in the geographical distribution of ICUs, in resource availability, and staffing patterns.
- A registry-based approach may offer a mechanism for continual evaluation of the epidemiology and serve as a platform for research and quality improvement.

## What are the new findings?

- The Indian Registry of IntenSive care (IRIS) was established in January 2019 as a cloud-based platform for case-mix evaluation, for benchmarking quality indicators and to serve as a platform for multi-centre critical care research and quality improvement.
- The registry currently includes 15 ICUs and has logged over 8000 patient encounters in 15 months. Nearly a quarter of patients admitted to these ICUs needed mechanical ventilation and the crude mortality was 9%
- Data availability for most parameters was above 95%.

## What do the new findings imply?

- A registry-based approach is feasible and can provide continual and high-quality information on critical illness, resource utilization and outcomes across ICUs in India.
- IRIS has provided a framework for navigating regulatory approvals, for data security and safety and for a sustainable funding model in India.
- The registry will serve as a platform for multi-centre observational and interventional research and quality improvement. Several such projects are already underway or being planned.

## Introduction

In India and other lower-middle-income countries (LMICs), the epidemiology of critical illness is distinct from high-income countries (HICs) in the types of diseases that bring patients into intensive care units (ICUs), in resource availability and access to care, in funding models for healthcare and in the burden of antimicrobial resistance. However, the published data on epidemiology of critical illness in India are limited. Most information currently comes from the INDICAPS multi-centre cross-sectional point prevalence study ^1^. There is also limited information on geographical spread, resource availability and staffing patterns across ICUs in India.

In contrast to a point prevalence study, an ongoing ICU patient registry provides a continual evaluation of service provision, epidemiology and quality of care. Well-established examples exist in the UK (Intensive Care National Audit and Research Centre-ICNARC) ^2^, Australia/New Zealand (ANZICS Centre for Outcomes and Resource Evaluation-CORE) ^3^) and Sweden (Swedish Intensive care Registry-SIR) ^4^. Recently, registries have expanded to middle-income countries including Brazil ^5^ and Southeast Asia, where the Network for Improving Critical Care Systems and Training (NICST- https://nicst.com/) has collaboratively developed registries in Sri Lanka (approximately 100 ICUs) and in Pakistan (nearly 20 ICUs) ^6, 7^.

In January 2019, in collaboration with NICST, we established the Indian Registry of IntenSive care (IRIS), modelled along registries in neighbouring countries, to map capacity and describe case-mix and outcomes. Our objectives were to describe the geographical distribution and resource availability of ICU/high-dependency unit (HDU) facilities in India; to describe the epidemiology, course and outcomes of patients admitted to these critical care units, to provide regular quality reports to individual participant ICUs of the registry; and to enable multi-centre quality improvement and research using the registry as the platform. In this report, we describe the implementation process, preliminary results, opportunities for improvement, challenges and future directions.

## Methods

We designed and implemented a cloud-based registry, similar to registries in Sri Lanka and Pakistan. Details of these models have been previously published ^6, 7^. All adult and paediatric ICUs in India were eligible to join if they committed to entering data for patients admitted to these ICUs. We excluded neonatal ICUs. The registry was implemented in stages. In the first instance, ICUs and Intensive Care Physicians known to the Investigators from existing research collaborations were invited to join IRIS and the directors were invited to complete a survey of resources and capacity. Once they obtained local ethical and administrative clearances, a dedicated dashboard was created on the registry platform for data entry. Each unit was then provided with secure login credentials. Table 1 contains a list of all enrolled ICUs at time of publication.

**Table 1.** Units participating in IRIS.

Name of hospital	City, State	Type	Model of care ^1^	Teaching program Yes/No ^2^
Apollo Main Hospital	Chennai, Tamil Nadu	Private	Semi-closed	Y
IQRAA HOSPITAL	Calicut, Kerala	Trust	Open	Y
Apollo Speciality Hospital - OMR	Chennai, Tamil Nadu	Private	Semi-closed	Y
Apollo Cancer Institute	Chennai, Tamil Nadu	Private	Semi-closed	Y
Apollo First Med Hospital	Chennai, Tamil Nadu	Private	Open	Y
Apollo Specialty Hospital, Vanagaram	Chennai, Tamil Nadu	Private	Semi-closed	Y
Apollo Childrens Hospital	Chennai, Tamil Nadu	Private	Closed	Y
Mehta Hospital	Chennai	Private	Semi-closed	N
Pushpagiri Medical College and Hospital	Thiruvalla, Kerala	Private	Closed	Y
Nanjappa Multi-specialty Hospital	Shimoga, Karnataka	Private	Semi-closed	N
All India Institute of Medical Sciences	Bhubaneswar, Odisha	Government	Semi-closed	Y
Ispat General Hospital	Rourkela, Odisha	Government	Open	N
Eternal Hospital	Jaipur, Rajasthan	Private	Semi-closed	Y
ABC Hospital	Vishakapatnam, Andhra Pradesh	Private	Semi-closed	N
Apollo Proton Cancer Centre	Chennai, Tamil Nadu	Private	Semi-closed	N

^1^Open = Intensive care physician consults, but does not direct care; Closed= Intensive care physician directs care and seeks additional consultation from other specialists as required; Semi-closed= hybrid of open and closed models. ^2^ Availability of teaching programs such as Indian Diploma in Critical Care Medicine, Fellowship of the National Board.

### Funding

IRIS was established with existing local resources in each of the ICUs without any external funding; NICST provided free access to the registry platform and technical support. Local IT support and server costs were borne by the critical care group at Apollo Hospitals ^8^. From November 2019, expansion of the registry has been supported by partial funding from the Wellcome Trust and the Mahidol Oxford Tropical Research Unit.

### Data collection and analysis

**Figure 1.**
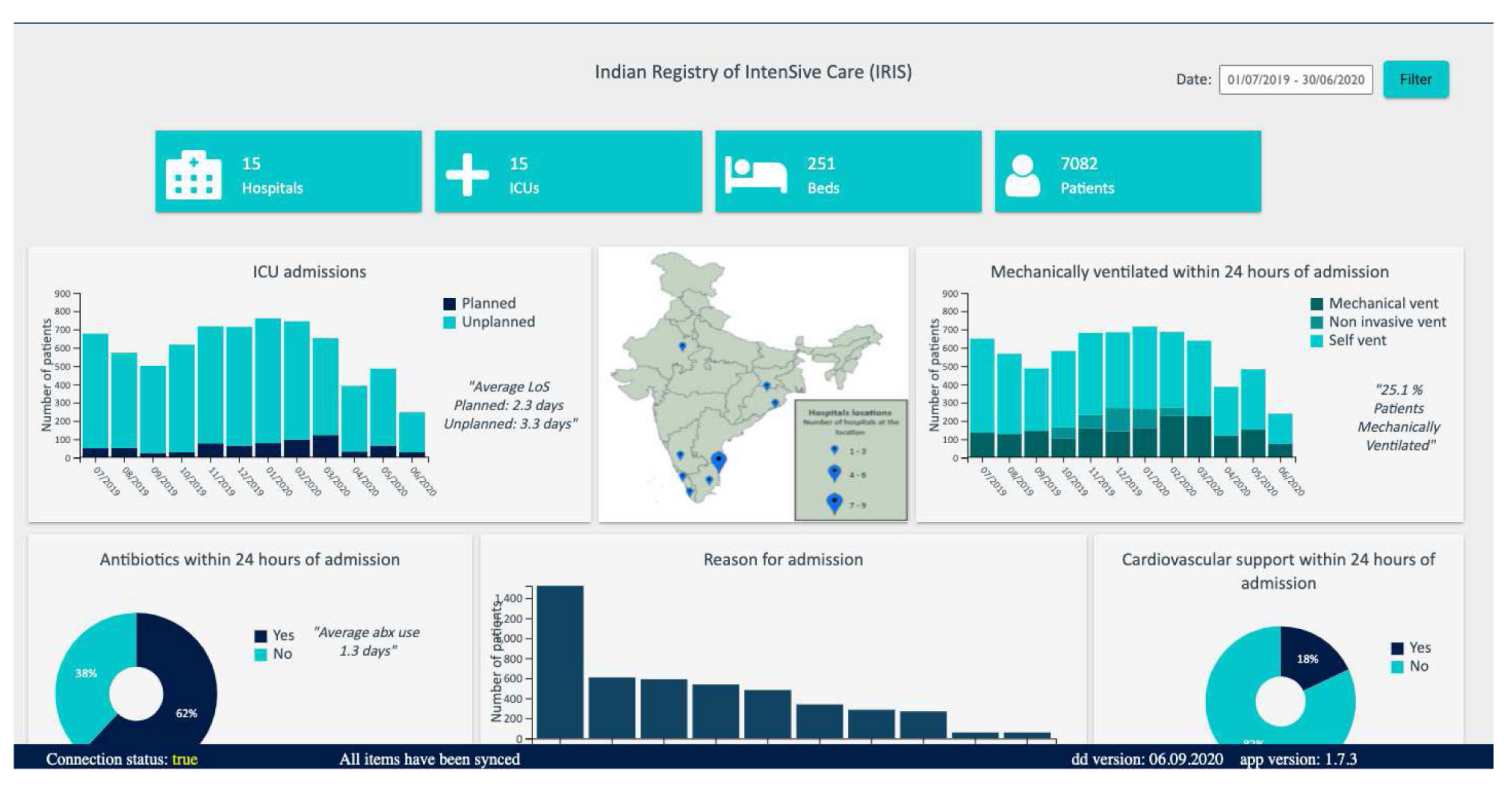
Dashboard view of the registry platform: aggregate view.

**Figure 2.**
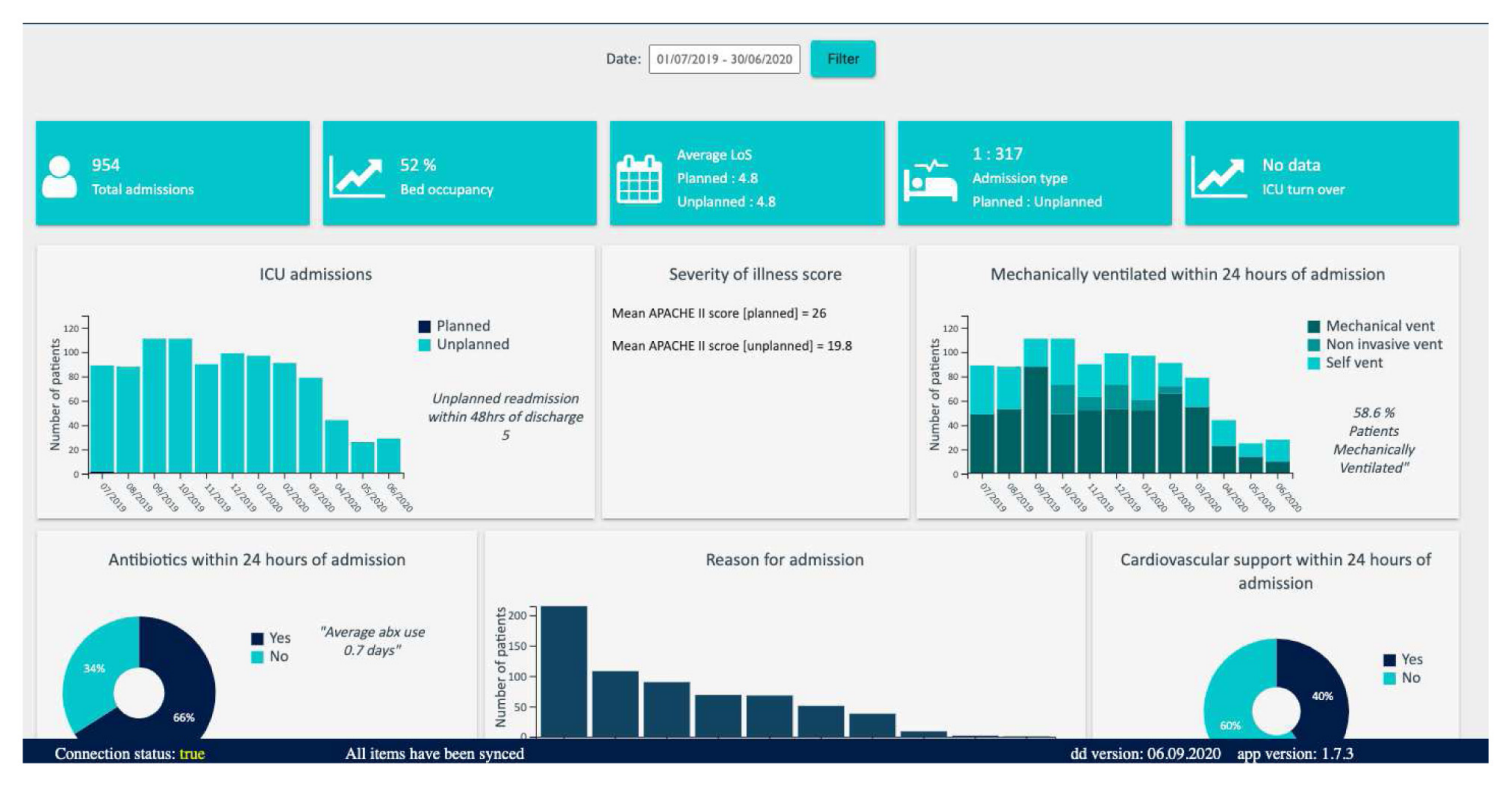
Dashboard view of the registry platform: unit-level view.

Data are collected by a designated representative (Physician, Physician Assistant, Registered Nurse, or a Research Assistant) through the electronic data collection platform of the registry ( Figure 1 and Figure 2). The registry has a minimum core dataset and an extended dataset for quality indicators. To manage data collection requirements, the minimum dataset is restricted to demographic variables (e.g. age, sex etc.), reasons for ICU admission (mapped as per APACHE IV ^9^ system and the SNOMED CT ^10, 11^ system), indicators of illness severity (e.g. need for mechanical ventilation, vasopressors etc), and ICU outcomes (e.g. ICU mortality, length of stay etc.). The extended quality dataset includes variables for several commonly used quality indicators. All participating ICUs collect the minimum core dataset; the quality indicator dataset is optional. The registry platform allows for paper data collection followed by entry onto the system as well as direct collection using a mobile application. Sites can choose either approach.

We use descriptive statistics to report our results. Categorical variables are reported as frequencies and percentages and continuous variables are reported as mean±SD or median and IQR based on distribution. We used Stata version 13.1 for all analyses (StataCorp. 2013. *Stata Statistical Software: Release 13.* College Station, TX: StataCorp LP.)

### Data quality

As per International standards ^12^, our data quality is focused on the elements of completeness, timeliness, consistency and validity. Completeness is checked monthly by an independent central data validator by comparing the number of admissions to the unit (using ICU census data obtained independently) against the number captured on the registry (due vs. captured numbers). We evaluate timeliness by assessing time from patient admission to data availability on the registry platform. Consistency over time is evaluated by examining for implausible trends in number of admissions, number of discharges and proportion of mechanically ventilated patients on a monthly basis. Validity is ensured by the logical flow of data (in sequential order, admission details, admission assessment, quality indicator data and discharge information). The platform’s existing internal data quality mechanisms – field completeness, value range validity, and branching logic – mean that users are immediately alerted to a potentially implausible or impossible value. Completeness for aggregate forms and individual variables are then visible on descriptive analytic dashboards. The completeness is reviewed weekly by the national registry teams, and the site leads.

### Data storage, access and security

IRIS hosts data on a secure cloud-based server; the front-end view to each ICU is shown in Figure 1 and Figure 2. Access to server is restricted to designated personnel and is protected with standard firewall and a valid secure socket layer (SSL) certificate. Each participating ICU owns and has access to its own data. In each ICU, the data entry executive and the ICU director are provided with unique login information and access is restricted to these personnel. All participating units have access to de-identified network-wide aggregate data which enables benchmarking and comparison.

### Reporting and benchmarking

Currently, units are able to download and print monthly reports of performance- this includes data on demographics, illness severity at admission as well as on outcomes (for units collecting the minimal dataset). For units collecting additional information on quality indicators, this information will also be available on their monthly reports. Of note, the reports can be downloaded or printed with several flexible time filters ( i.e. monthly, quarterly, yearly etc.)

Additionally, every unit has access to the aggregate data dashboard for comparison of overall registry performance versus their own unit’s performance. 

We are not, at this point in time, sharing comparative reports between units or highlighting outliers or prescribing steps for improvement. The main reason is that ours is a fledgling registry with units being onboarded gradually over time. The idea of a critical care registry and its objectives and goals are novel to several units and ICU clinicians in India. Additionally, not all units collect information on quality indicators and this is presently an optional form. While the ultimate goal is to move towards benchmarking and comparisons, our approach has been to proceed slowly and with caution in order to ensure and sustain buy-in from the stakeholders. The transition to a full clinical quality registry with reporting and benchmarking of resources, processes, and outcomes will be a decision taken by the IRIS steering committee, with input from all the contributing ICUs.

### Ethics and patient consent

As the primary purpose of the registry is evaluation of case-mix and outcomes, each ICU was asked to consult their local regulatory teams and obtain ethical and administrative clearances as mandated by their respective sites. At some ICUs this meant both ethics committee and hospital administrative approvals and at other sites this meant only the need for administrative approval.

Internationally, registries do not obtain individual patient consent for registries as the primary purpose of the registry is evaluation of case-mix, quality and service provision. The need for individual patient level consent would make the concept of a registry untenable ^13^. Alternatives to individual consent include a waiver of consent (if approved by the Ethics committee), display of information about the registry in the ICU with an option for opt-out (ICNARC model) ^14^, or modification of the general critical care consent form to add a clause on routine data collection for audit and quality improvement purposes. Most ICUs in IRIS have taken the last approach.

### Governance structure, research and authorship policies

IRIS is overseen by a steering committee with national and international members with specific expertise in registries and in the delivery of critical care in resource limited settings. All major decisions on the vision and direction of IRIS are approved by the steering committee. IRIS also has an operations team that oversees day-to-day functioning of the registry. Additionally, a coordinating committee has members from all participating ICUs to ensure their views are well represented. In addition, for any research derived from IRIS data, a separate ethics committee approval is essential from all the participating sites. IRIS also has clearly outlined guidelines for authorship for publications arising out of the collaborative.

## Results

**Figure 3.**
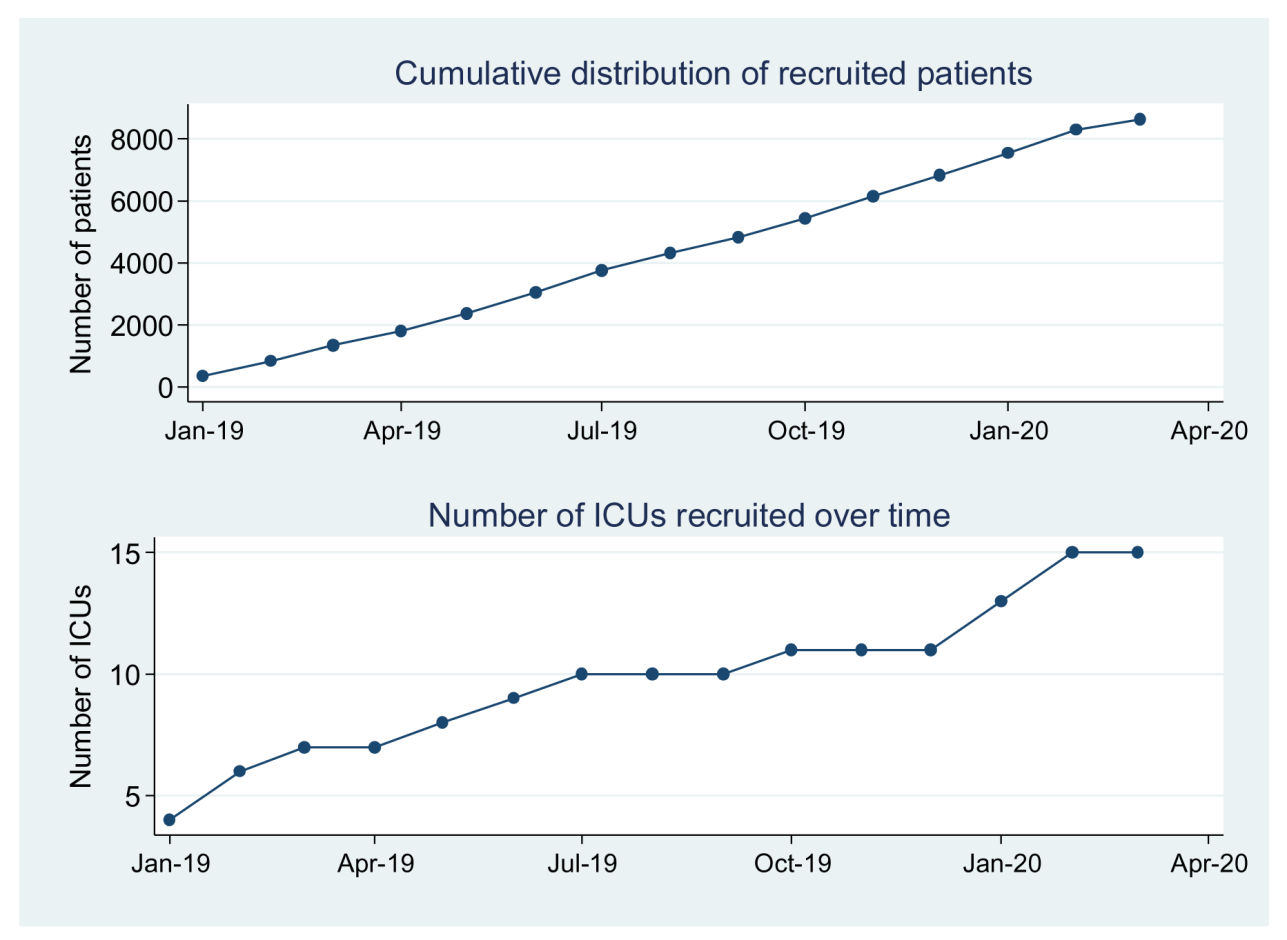
Recruitment of ICUs and patients over time.

**Figure 4.**
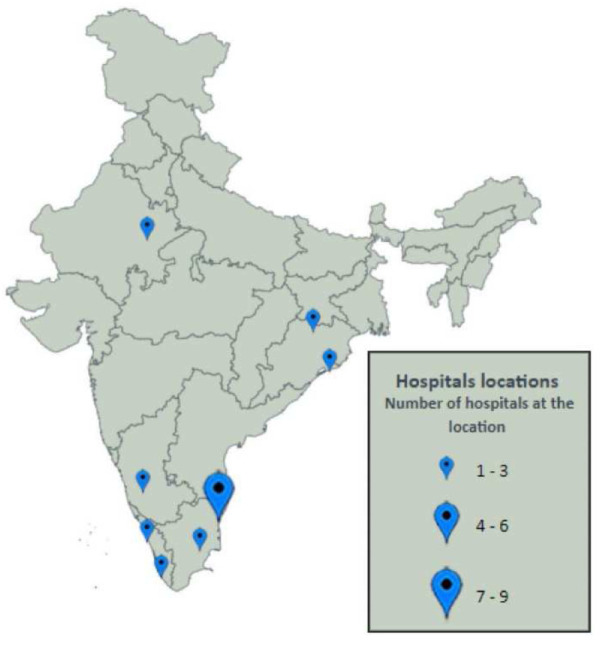
Distribution of the participating ICUs.

The registry was established in January 2019 and currently includes 14 adult and 1 paediatric ICU in the network (232 adult ICU beds and 9 paediatric ICU beds; Figure 3 and Figure 4, Table 1). None of the ICUs that we approached declined participation and in 2019, we added approximately 3 ICUs to the registry every quarter. Table 2 describes patient characteristics as of 15 ^th^ March 2020; enrolment over time is shown in Figure 3. There have been 8721 patient encounters with a mean age of 56.9 (SD 18.9); 61.4% of patients were male and admissions to participating ICUs were predominantly unplanned (87.5%). The most common reason for admission was cardiovascular (using APACHE IV classification) in 24.5% of patients. At admission, most patients (61.5%) received antibiotics, 17.3% needed vasopressors, and 23.7% were mechanically ventilated. Mortality for the entire cohort was 9%. Table 3 describes the completeness of information for the registry variables. Demographics, clinical parameters, and indicators of admission severity had an availability of more than 95%. Among the laboratory parameters, blood urea had the lowest availability (81%).

**Table 2.** Patient characteristics (data updated to 15 ^th^ March 2020).

Characteristics	Patients, n (%) (N= 8721)
**Age-**mean (SD) in years	56.9 (18.9)
**Gender** Male Female	5355 (61.4) 3366 (38.6)
**Admission Type** Planned Unplanned	1084 (12.4) 7637 (87.6)
**Ventilation status at admission** Non-ventilated Mechanically ventilated Non-invasive ventilated Not Recorded	5960 (68.3) 2066 (23.7) 408 (4.7) 287 (3.3)
**Number of vasopressors at admission** 1 2 More than 2 None Not Recorded	1093 (12.5) 299 (3.4) 119 (1.4) 6922 (79.3) 288 (3.3)
**Antibiotics on admission** Yes No Not Recorded	5333 (61.1) 3085 (35.4) 3030 (3.5)
**Sedated on admission** Yes No Not Recorded	1318 (15.1) 7127 (81.7) 276 (3.2)
**Reason for admission (APACHE IV** **Coding)** Cardiovascular diagnosis Neurologic Respiratory Gastrointestinal Genitourinary Metabolic/Endocrine Trauma Haematology Trauma Musculoskeletal/Skin Cardiac surgery Transplant Not Recorded	2070(23.7) 1787 (20.5) 1448 (16.6) 990 (11.3) 686 (7.9) 471 (5.4) 403 (4.6) 400 (4.6) 355 (4.0) 67(0.8) 13 (0.1) 29 (0.3)
**Overall APACHE II score** **mean(SD)**	20.0(6.9)
**Status at ICU discharge** Alive Dead In ICU Not Recorded	7591 (87.0) 788 (9.0) 324 (3.7) 18 (0.2)

**Table 3.** Availability of variables on the IRIS dataset.

Parameters	n (%) (n=8721)
Age	8696 (99.7)
Gender	8721 (100)
Admission type	8721 (100)
Glasgow Coma Scale	8442 (96.8)
Diagnosis type	8706 (99.8)
Primary system	8692 (99.7)
Mechanically ventilated on admission	8434 (96.7)
Vasoactive drugs on admission	8433 (96.7)
Use of antibiotics on admission	8418 (96.5)
Sedated on admission	8445 (96.8)
Heart rate	8438 (96.7)
Systolic blood pressure	8448 (96.9)
Diastolic blood pressure	8448 (96.9)
Respiratory rate	8444 (96.8)
Temperature	8323 (95.4)
Haemoglobin	8096 (92.8)
Blood urea	7068 (81.0)
White blood cells	7773 (89.1)
Platelet	7765 (89.0)
Survival status at ICU discharge (from discharged patients (N=8397))	8379 (99.8)

Figure 3 depicts enrolment of the patients and ICUs over time. On average, we were able to recruit 3 or 4 ICUs and approximately 1600 patients each quarter. Table 4 contains a profile of the participating ICUs.

**Table 4.** Profile of the participating units.

Characteristics	n (%) (n=15)
**Institution category** Government Private Trust	2 (13.3) 12 (80.0) 1 (6.7)
**Model of care** Open Closed Semi-closed	5 (33.3) 2 (13.3) 8 (53.4)
Teaching Program No Yes	7 (47.6%) 8 (53.3%)
**ICU consultant primary specialty** Anaesthesia Medicine Pulmonology	8 (53.4) 5 (33.3) 2 (13.3)
**1:1 Nursing of ventilated patients during day** Yes	12 (80.0)
**1:1 Nursing of ventilated patients during night** Yes	12 (80.0)
**Healthcare assistants and technicians** Yes	13 (86.7)
**Physiotherapist** Yes	10 (66.7)
**Radiology technician for portable x-ray** Yes	15 (100)
**Backup automatic electricity generator** Yes	15 (100)
**Hand washing facilities in the intensive care** **unit** Yes	15 (100)
**isolation Rooms** Yes	13 (86.7)
**Access to arterial blood gas analysis** Yes	15 (100)
**Access to external internet** Yes	14 (93.3)
**Telephone (Direct)** Yes	11 (73.3)

## Discussion

We have demonstrated feasibility of a registry of unselected patients admitted to ICUs to describe near real-time the case-mix and outcomes from an emerging ICU network in India. In a period spanning 15 months, we were able to enrol 15 ICUs and collect information on basic epidemiology of critical illness from these participating units.

There are several important lessons from our approach. IRIS provides a template for a sustained and extended period of multi-centre collaboration. Although we are currently restricted to tracking case-mix and outcomes, valuable for epidemiological information, the potential for extending the network to lead quality improvement work and enable multi-centre research is clear. Such additions would be invaluable during the current Coronavirus Disease 2019 pandemic, where a pre-existing network can be far nimbler and timelier in collecting additional data fields relevant to a pandemic and in testing therapies. In non-pandemic periods, several examples of research priorities for India and the broader region exist, which are amenable to being answered by a collaborative approach exemplified by IRIS. Such examples include, but are not restricted to, epidemiology of critical illness related to tropical infections, the impact of multi-drug resistant organisms on outcomes from critical illness, and epidemiology of locally relevant non-infectious pathologies such as snake bites and organophosphorous poisoning.

IRIS has also overcome key ICU and hospital level hurdles to the establishment of a registry-based network. Important steps to successful deployment of the registry across ICUs have included a flexible approach to informed consent while being fully adherent to the requirements of ethics committees, and frequent and meaningful stakeholder engagement. The registry’s platform has several features that have facilitated participation, including a small core dataset to limit the burden of data collection, the availability of ICU-level real-time information on case-mix and outcomes on a dashboard, and the option of mobile data collection to avoid the need for double data entry. Stakeholder engagement has identified existing motivated personnel (registered nurses, physician assistants or research assistants) in the participating ICUs to contribute a small portion of time to collect data, with academic (acknowledgement or authorship in research papers) and modest financial incentives for these professionals, where feasible. This approach has addressed the challenges of data collection and entry.

Notwithstanding early success, the implementation of an ambitious multi-centre critical care registry in a highly heterogenous and diverse country such as India comes with several key challenges, described below.

*Managing data burden*: Registries must balance the need for granular data to maximise usefulness of collected information against the competing burden of data collection. IRIS attempts to achieve this balance by having a minimum core dataset mandatory for all participating ICUs and an extended quality dataset for ICUs with additional resources. The minimum dataset provides useful unit-specific information and benchmarking data, and our experience has been that participating units value the information. We constantly review the variables that constitute the core dataset to decide on the need for revisions based on perceived usefulness.

*Human resources*: As with all resource-limited settings, availability of data collection personnel is a challenge. As described, we have mitigated this by enabling professionals with different backgrounds to function as part-time data collectors. Challenges, however, remain in sustaining motivation and ongoing engagement. These challenges are expected to be more severe at publicly funded government hospitals.

*Other challenges*: These include engaging front-line clinicians in the use of the collected data and addressing misgivings about data confidentiality from potential new participants. We address these issues through ongoing engagement with all stakeholders through regular communication using formal and informal electronic technologies such as email and ‘WhatsApp’ groups.

### Next steps

The vision of IRIS is ambitious and there are several planned next steps. In addition to expansion of the registry, the short-term target is to expand quality indicators on the registry platform. Units motivated to collect such data will additionally have access to unit quality indicators and can benchmark to the wider aggregate indicator information from the registry.

The crude mortality in the registry is 9%, and less than a quarter of patients received mechanical ventilation even though the bulk of admissions were unplanned. This could be explained by the mix of high-dependency and intensive care-level units in the registry. As a next step, we are developing a contextually relevant risk-adjustment model which will aid in benchmarking of participating units.

A series of multi-centre registry-embedded research projects are being designed/planned and we hope to complete some of these studies by the mid-2021. IRIS is a founding member of the Critical Care Asia network, a Wellcome Trust-supported network of critical care registries across South and South-East Asia ^15^. All these registries operate on the same platform as IRIS and will harmonize data collection and analysis, opening avenues for data sharing of deidentified information and in serving as a mechanism for multi-country, context-appropriate, critical care research in South and South-East Asia. IRIS is a contributor to LOGIC ^16^- an international collaborative of registries and is preparing to participate in the registry embedded REMAP-CAP ^17, 18^ adaptive trial for COVID-19 patients.

## Conclusion

IRIS represents a successful model for the continual evaluation of critical illness epidemiology in India and provides a framework for the deployment of multi-centre quality improvement and context-relevant clinical research studies for the critical care community in India.

## Data availability

Pooled data from IRIS are available from the IRIS Dashboard at https://nicst.com/picu-iris-public/.

The IRIS collaboration supports and welcome data sharing. Raw data will be made available to qualified researchers who provide a detailed and methodologically sound proposal with specific aims that are clearly outlined. Such proposals will be screened by the IRIS Steering committee for approval. Data sharing will be for the purposes of medical research and under the auspices of the consent under which the data were originally gathered.

To gain access, qualified researchers will need to sign a data sharing and access agreement and will need to confirm that data will only be used for the agreed upon purpose for which data access was granted. Researchers can contact the corresponding author through electronic mail ( bharath@icuconsultants.com) for such access; alternatively, IRIS can be contacted at info@irisicuregistry.org and joinus@irisicuregistry.org.

## Author information

All authors are affiliated with the Indian Registry of IntenSive care (IRIS). Authors are listed in alphabetical order.
