# Supplementary material for: Implementing an intensive care registry in India: preliminary results of the case-mix program and an opportunity for quality improvement and research
Source: Wellcome Open Res. 2020 Oct 21;5:182. Originally published 2020 Jul 30. [Version 2] doi: 10.12688/wellcomeopenres.16152.2 (PMC7642994; doi:10.12688/wellcomeopenres.16152.2)
Supplement: Peer review history

# Peer review: Reviewer response for version 2

**DOI:** 10.21956/wellcomeopenres.18041.r41021

**Published:** 2020-11-04

**Authors:** David Pilcher

**Affiliations:** 1 Australian and New Zealand Intensive Care Research Centre, Monash University, Melbourne, Australia; 2 Department of Intensive Care, Alfred Health, Melbourne, Australia

Thanks for sending through the revisions. I’ve enjoyed reading the paper and am happy with the changes made by the authors. All the best with the publication.

Is the work clearly and accurately presented and does it cite the current literature?

Yes

If applicable, is the statistical analysis and its interpretation appropriate?

Partly

Are all the source data underlying the results available to ensure full reproducibility?

Partly

Is the study design appropriate and is the work technically sound?

Yes

Are the conclusions drawn adequately supported by the results?

Yes

Are sufficient details of methods and analysis provided to allow replication by others?

Partly

Reviewer Expertise:

Critical Care Medicine, Epidemiology and Registry science, Transplantation and Organ Donation.

I confirm that I have read this submission and believe that I have an appropriate level of expertise to confirm that it is of an acceptable scientific standard.

# Peer review: Reviewer response for version 2

**DOI:** 10.21956/wellcomeopenres.18041.r41022

**Published:** 2020-10-22

**Authors:** Lowell Ling

**Affiliations:** 1 Department of Anaesthesia and Intensive Care, The Chinese University of Hong Kong, Hong Kong, Hong Kong

The authors have addressed all of my concerns. This initiative is particularly inspiring because it strives to be inclusive and has managed to include a range of private and public ICUs. Congratulations and hope to see more work from this registry.

Is the work clearly and accurately presented and does it cite the current literature?

Yes

If applicable, is the statistical analysis and its interpretation appropriate?

Not applicable

Are all the source data underlying the results available to ensure full reproducibility?

Yes

Is the study design appropriate and is the work technically sound?

Yes

Are the conclusions drawn adequately supported by the results?

Yes

Are sufficient details of methods and analysis provided to allow replication by others?

Yes

Reviewer Expertise:

Critical care, sepsis.

I confirm that I have read this submission and believe that I have an appropriate level of expertise to confirm that it is of an acceptable scientific standard.

# Peer review: Reviewer response for version 1

**DOI:** 10.21956/wellcomeopenres.17732.r39805

**Published:** 2020-10-05

**Authors:** David Pilcher

**Affiliations:** 1 Australian and New Zealand Intensive Care Research Centre, Monash University, Melbourne, Australia; 2 Department of Intensive Care, Alfred Health, Melbourne, Australia

Thank you for asking me to review this paper which describes the development and initial results from the implementation of the Indian critical care registry. The paper describes the activity and outcomes from 14 adult ICUs and 1 paediatric ICU reporting to the registry between January 2019 and March 2020. Basic demographics of nearly 9000 patients are reported, of whom 61% were male, most admissions were unplanned and 9% died.

 The paper is of interest and provides useful information that is relevant for any country which might consider starting such a registry. The authors provide objective data in addition to subjective learnings which are relevant not only to those from low and middle income countries but also to areas with well-developed health care systems. The paper is clearly written and appropriately analysed. There are many practical tips such as the flexible approach to consent/participation permissions which should be commended.

Main comments

**1. Comparative reporting**

 One of the most important purposes of a clinical quality registry is reporting and benchmarking of resources, processes and outcomes. To me, this is much more important than any pure research output from a registry. Reporting should be covered in greater depth, perhaps in its own additional separate section to go with the others already presented in the Results. Dashboard figures of individual site information and of aggregated whole of registry information are provided but it is unclear how comparisons between units/types of patients/etc are reported? How are comparative outcomes reported presently? What are the registry’s plans for doing this in future? Is there an ‘outlier process’ if sites appear to statistically different outcomes compared to others? The authors note a need for a local risk adjustment model. How is severity of illness presently reported? (see comment below too).

 My opinion is that comparative reporting of outcomes, when done well with robust risk-adjusted statistical analysis within an appropriate governance framework involving clinicians, registry personnel and policy makers/health department representatives, has great capacity to improve patient outcomes. The authors appear to skirt over this topic. Do they think discussion of it will impact participation in the registry? Do the authors think negative perceptions about reporting of comparative performance are any more or less of a problem in India than in other countries?

**2. Clarify Mortality**

 Clarify the mortality outcomes reported. Are the authors reporting in-ICU mortality or in-hospital mortality? Appears to be the former. If so, why isn’t in-hospital mortality considered and is there any intention to measure longer term survival or functional outcomes?

**3. Additional compliance information and implications for ‘representative-ness’ of contributing ICUs**

 High compliance of physiological and biochemical variables is commendable. How many of these ICUs have electronic medical record systems which can provide the data? Do you have compliance information for process, diagnosis or chronic health information which usually require some form of manual data entry or at least checking?

 It is very interesting (but sobering to someone working in a high-income country) to see that ICUs are asked to state if they have a back-up generator, hand washing facilities, internet or telephone (all of which would be assumed in my country). The fact that these questions are asked alone and that these services are almost all available, suggests that there are ICUs in India without these resources. How do the authors propose to include these under resourced ICUs (where monitoring of patients’ outcomes may be even more important)?

**4. Clarify ICUs vs Hospitals.**

 Are these hospitals with one ICU providing all services for the hospital (common in UK & ANZ) or speciality ICUs of which there may be multiple within a hospital (predominant US model)? Please provide breakdown of ICU speciality type if possible, in Table 1.

 India is a big place! Do the authors have any indication of the total number of hospitals and ICUs in the country? They describe a great start with rapidly increasing participation. How much further is there to go?

**5. Clarify funding and payment characteristics.**

 Although the funding for the registry’s administrative functions and operational activities are described it is not clear how this works for the sites themselves. Funding models for registries vary hugely throughout the world. Do the contributing sites pay to participate? Is funding provided to sites for data collection? Is site the model for participation the same for both public and private sector? Does this vary depending on whether the site is associated with Apollo who also appear to be providing registry infrastructure support?

Minor comments

 Table 2- Please provide overall severity of illness with APACHE II and IV scores, since you appear to be recording and reporting these (at least APACHE II on the figure). Do the authors report predicted mortality from these scoring systems? Are these appropriate metrics to report for Indian ICUs? See above comment also.

 Table 2 - Please provide the CVS group broken into cardiac surgery and other cardiovascular diagnoses.

 Cut any superfluous acronyms e.g. MCD.

 Figure 3 just looks like a STATA bog standard. Please state if you are using a stats package to either report the paper’s results or have this embedded within the registry reporting system. Please tidy up the formatting of the figure.

 I thank the authors for the opportunity to review this paper, commend them for their work and for producing an informative and interesting paper. I look forward to seeing further outputs from the registry as it continues to grow.

Is the work clearly and accurately presented and does it cite the current literature?

Yes

If applicable, is the statistical analysis and its interpretation appropriate?

Partly

Are all the source data underlying the results available to ensure full reproducibility?

Partly

Is the study design appropriate and is the work technically sound?

Yes

Are the conclusions drawn adequately supported by the results?

Yes

Are sufficient details of methods and analysis provided to allow replication by others?

Partly

Reviewer Expertise:

Critical Care Medicine, Epidemiology and Registry science, Transplantation and Organ Donation.

I confirm that I have read this submission and believe that I have an appropriate level of expertise to confirm that it is of an acceptable scientific standard, however I have significant reservations, as outlined above.

# Response

**Published:** 2020-10-19

**Authors:** IRIS India

**Affiliations:** Indian Registry of IntenSive care, India

We would like to express our sincere appreciation and gratitude for the reviewer feedback. We address their comments below.

Reviewer 2: Dr David Pilcher

**1. Comparative reporting : One of the most important purposes of a clinical quality registry is reporting and benchmarking of resources, processes and outcomes. To me, this is much more important than any pure research output from a registry. Reporting should be covered in greater depth, perhaps in its own additional separate section to go with the others already presented in the Results. Dashboard figures of individual site information and of aggregated whole of registry information are provided but it is unclear how comparisons between units/types of patients/etc are reported? How are comparative outcomes reported presently? What are the registry’s plans for doing this in future? Is there an ‘outlier process’ if sites appear to statistically different outcomes compared to others? The authors note a need for a local risk adjustment model. How is severity of illness presently reported? (see comment below too).**

**My opinion is that comparative reporting of outcomes, when done well with robust risk-adjusted statistical analysis within an appropriate governance framework involving clinicians, registry personnel and policy makers/health department representatives, has great capacity to improve patient outcomes. The authors appear to skirt over this topic. Do they think discussion of it will impact participation in the registry? Do the authors think negative perceptions about reporting of comparative performance are any more or less of a problem in India than in other countries?**

 We would like to thank the reviewer for raising several important issues:

Currently, units are able to download and print monthly reports of performance- this includes data on demographics, illness severity at admission as well as on outcomes (for units collecting the minimal dataset). For units collecting information on quality indicators, this information will also be available on their monthly reports. Of note, the reports can be downloaded and printed with several flexible time filters ( i.e. monthly, quarterly, yearly etc.) 

Additionally, every unit has access to the aggregate data dashboard for comparison of overall registry performance versus their own unit’s performance.

We are not, at this point in time, sharing comparative reports between units or highlighting outliers or prescribing steps for improvement. The main reason is that ours is a fledgling registry with units being onboarded gradually over time. The idea of a critical care registry and its objectives and goals are novel to several units and ICU clinicians. Additionally, not all units collect information on quality indicators and this is presently an optional form/s. While the ultimate goal is to move towards benchmarking and comparisons, our approach has been to proceed slowly and with caution in order to ensure and sustain buy-in from the stakeholders.  The transition to a full clinical quality registry with reporting and benchmarking of resources, processes, and outcomes will be  a decision taken by the IRIS steering committee, with input from all the contributing ICUs.

Severity of illness is currently reported using the APACHE II framework. We started collecting data on variables required for APACHE II only from the month of 07/2019 In addition, we have developed and validated a simplified illness severity scoring system called the e-TropICS ( under review with PLOS One- link to preprint: https://www.researchsquare.com/article/rs-53555/v1 ) using universally available variables. Our plan is to display both the APACHE II and the e-TropICS on the registry platform in the interim and once we refine calibration for the e-TropICS model, migrate entirely to this. 

**We have now added a section on ‘reporting and benchmarking’ to ‘Methods’**

**2. Clarify Mortality**

**Clarify the mortality outcomes reported. Are the authors reporting in-ICU mortality or in-hospital mortality? Appears to be the former. If so, why isn’t in-hospital mortality considered and is there any intention to measure longer term survival or functional outcomes?**

We are reporting ICU mortality in the registry- this was chosen initially as a pragmatic approach to minimise data burden capture. However, we have incorporated hospital mortality and post hospital discharge outcomes (quality of life etc.) as optional fields in the subsequent iterations of the platform. The goal is to move towards 100% data collection for hospital outcomes. 

**Clarified in the section ‘Data collection’ – line number 7.**

**3. Additional compliance information and implications for ‘representative-ness’ of contributing ICUs**

**High compliance of physiological and biochemical variables is commendable. How many of these ICUs have electronic medical record systems which can provide the data? Do you have compliance information for process, diagnosis or chronic health information which usually require some form of manual data entry or at least checking?**

**It is very interesting (but sobering to someone working in a high-income country) to see that ICUs are asked to state if they have a back-up generator, hand washing facilities, internet or telephone (all of which would be assumed in my country). The fact that these questions are asked alone and that these services are almost all available, suggests that there are ICUs in India without these resources. How do the authors propose to include these under resourced ICUs (where monitoring of patients’ outcomes may be even more important)?**

None of the participating ICUs have electronic health records. We perform random source data verification of records at sites as part of our validation exercise. 

In the manuscript, under the  ‘Data Quality’ section, we have highlighted all the steps we currently take to ensure quality.

Although we do not have data on either the total number of ICUs across India or information on number of ICUs that lack such facilities – and neither does the central or any state government – we are aware that several district hospital ICUs lack such basic facilities. 

Our approach to including ICUs in the registry has been pragmatic- we have only approached ICUs and Intensive Care Physicians that are either well known to the investigators or have participated in previous research collaborations and as such were perceived to either have the interest or commitment to this initiative. Our approach has been a gradual, but concerted, effort at adding ICUs over time. For 2019, we added approximately 3 ICUs to the registry every quarter.

Our vision is to be inclusive and diverse in the representation of units on the registry and we will attempt to include some of these resource-constrained units as we expand. Our registry very much remains a work in progress.

**4. Clarify ICUs vs Hospitals.**

**Are these hospitals with one ICU providing all services for the hospital (common in UK & ANZ) or speciality ICUs of which there may be multiple within a hospital (predominant US model)? Please provide breakdown of ICU speciality type if possible, in Table 1.**

**India is a big place! Do the authors have any indication of the total number of hospitals and ICUs in the country? They describe a great start with rapidly increasing participation. How much further is there to go?**

Some of the participating hospitals indeed have more than one ICU- for all participating IRIS hospitals, the mixed-medical surgical or the general ICU is the one that has been included in the registry. This has been the preference of the participating sites. At one of the sites ( AIIMS, Bhubaneswar), we have recently added a SARI-ICU as an additional unit. Eternal Hospital, Jaipur has expressed an interest in expanding to other ICUs in their hospital. As it stands, there are currently 14 adult hospitals with 15 ICUs represented ( one additional ICU from AIIMS, Bhubaneswar). From a registry standpoint, there is no restriction on the number of units allowed to participate from each hospital and we expect that additional units from each of the participating sites will join the registry in the future.

There is no official data on the number of ICUs across India. In a recent manuscript, we describe ICU capacity and resources based on information from different sources ( https://journals.sagepub.com/doi/full/10.1177/1751143720952590). Our aim is to add as many units as feasible and within the limits of funding support that we can provide. Additionally, as stated above, we are aiming for diversity and representativeness as we expand. 

**5. Clarify funding and payment characteristics.**

**Although the funding for the registry’s administrative functions and operational activities are described it is not clear how this works for the sites themselves. Funding models for registries vary hugely throughout the world. Do the contributing sites pay to participate? Is funding provided to sites for data collection? Is site the model for participation the same for both public and private sector? Does this vary depending on whether the site is associated with Apollo who also appear to be providing registry infrastructure support?**

No, the contributing sites do not have to pay anything. The only requirement is a commitment to ongoing data collection and participation in registry activities. We provide support for a data collector (either part-time or full-time) and for IT support ( tablet PC, internet connection, if required, printer etc.). Yes, the model for participation is same for public and private hospitals and there is no preferential treatment for any site.

Funding for the coordinating centre ( Apollo Main Hospital, Chennai) includes support for a National Coordinator, Implementation Manager, Validation Officer, and support for office expenses ( in addition to what has been described in the previous paragraph).

**Minor comments**

**Table 2- Please provide overall severity of illness with APACHE II and IV scores, since you appear to be recording and reporting these (at least APACHE II on the figure). Do the authors report predicted mortality from these scoring systems? Are these appropriate metrics to report for Indian ICUs? See above comment also.**

APACHE II information added to Table 2. Regarding illness severity and predicted mortality, we have addressed this in response to comment number 1.

**Table 2 - Please provide the CVS group broken into cardiac surgery and other cardiovascular diagnoses.**

We have included this now.

**Cut any superfluous acronyms e.g. MCD**

Removed.

**Figure 3 just looks like a STATA bog standard. Please state if you are using a stats package to either report the paper’s results or have this embedded within the registry reporting system. Please tidy up the formatting of the figure.**

We used Stata 13.1 and we have now added this in our section on data quality. We have reformatted the figure.

# Peer review: Reviewer response for version 1

**DOI:** 10.21956/wellcomeopenres.17732.r39791

**Published:** 2020-08-03

**Authors:** Lowell Ling

**Affiliations:** 1 Department of Anaesthesia and Intensive Care, The Chinese University of Hong Kong, Hong Kong, Hong Kong

The authors describe the establishment of Indian Registry of IntenSive care (IRIS), a multicentre ICU registry in India. They provide the rationale for starting a registry and describe a vision for the potential for growth and utility of a registry. Although it was only started in January 2019, it has already recorded over 8000 patient encounters. They described the participating units and availability of laboratory and clinical data. They also provided some early data on patient characteristics admitted to ICUs in participating units.

 The authors should be commended on a fantastic initiative and well written manuscript. This is an important initiative that will provide invaluable insight into ICU delivery in low- and lower middle-income countries. I have no doubt it will become a great resource for benchmarking and facilitate clinical research.

 Some suggestions:

1. In the discussion the authors stated: "In non-pandemic periods, several examples of research priorities for India and the broader region exist, which are amenable to being answered by a collaborative approach exemplified by IRIS". > Please cite examples
2. Is there any data on how long it takes for one patient data entry?
3. Please specify how many times the registry had to clarify/check for implausible and impossible values and how this will be fed back to the person entering the data.
4. Please describe any units that declined participation and cite reasons why, this may help readers judge barriers to joining this initiative.

Overall I highly recommend this manuscript for indexing as it is an important step to establishing this important registry.

Is the work clearly and accurately presented and does it cite the current literature?

Yes

If applicable, is the statistical analysis and its interpretation appropriate?

Not applicable

Are all the source data underlying the results available to ensure full reproducibility?

Yes

Is the study design appropriate and is the work technically sound?

Yes

Are the conclusions drawn adequately supported by the results?

Yes

Are sufficient details of methods and analysis provided to allow replication by others?

Yes

Reviewer Expertise:

Critical care, sepsis.

I confirm that I have read this submission and believe that I have an appropriate level of expertise to confirm that it is of an acceptable scientific standard, however I have significant reservations, as outlined above.

# Response

**Published:** 2020-10-19

**Authors:** IRIS India

**Affiliations:** Indian Registry of IntenSive care, India

We would like to express our sincere appreciation and gratitude for the reviewer feedback. We address their comments below.

Reviewer 1: Dr Lowell Ling

1. **In the discussion the authors stated: "In non-pandemic periods, several examples of research priorities for India and the broader region exist, which are amenable to being answered by a collaborative approach exemplified by IRIS". > Please cite examples**

Examples have now been incorporated **( paragraph number 2 under ‘discussion’ – lines 10-13**)- the examples cited relate to critical illness resulting from tropical infections such as malaria, dengue, leptospira etc. , the impact of multi-drug resistant organisms on outcomes from critical illness in LMICs and strategies for alleviation, and epidemiology of non-infectious pathologies such as snake bite, specific local toxicology such as organophosphorous compound poisoning, among others.

2. **Is there any data on how long it takes for one patient data entry?**

From interviews evaluating registry implementation, interviewees report that data entry for new admissions takes 12-20 mins and daily entry each day and at discharge takes 5-10 mins.

3. **Please specify how many times the registry had to clarify/check for implausible and impossible values and how this will be fed back to the person entering the data.**

We do not audit auto alerts for individual variables. The platform’s existing internal data quality mechanisms – field completeness, value range validity, and branching logic, mean that users are immediately alerted to a potentially implausible or impossible value. Completeness for aggregate forms and individual variables are then visible on descriptive analytic dashboards. The completeness is reviewed weekly by the national registry teams, and the site leads.

*Information on this has now been added to the section ‘Data Quality’ (lines 10-14)*

**4. Please describe any units that declined participation and cite reasons why, this may help readers judge barriers to joining this initiative.**

None of the units that we have approached has declined participation. Having said this, we have only approached ICUs and Intensive Care Physicians that are either well known to the investigators or have participated in previous research collaborations and as such were perceived to either have the interest or commitment to this initiative. Our approach has been a gradual, but concerted, effort at adding ICUs over time. For 2019, we added approximately 3 ICUs to the registry every quarter.

*Information has been added to Methods (paragraph 1, lines 5 and 6) and Results (paragraph 1, lines 3 and 4).*
